# *E. coli* Induced Experimental Model of Primary Biliary Cirrhosis: At Last

**DOI:** 10.1155/2014/848373

**Published:** 2014-12-16

**Authors:** Andreas L. Koutsoumpas, Daniel S. Smyk, Dimitrios P. Bogdanos

**Affiliations:** ^1^Division of Medicine, School of Health Sciences, University of Thessaly, Thessaly, Viopolis, 40500 Larissa, Greece; ^2^Institute of Liver Studies, King's College London School of Medicine at King's College Hospital, Denmark Hill Campus, London SE5 9RS, UK; ^3^Cellular Immunotherapy and Molecular Immunodiagnostics, Biomedical Section, Institute of Research and Technology-Thessaly (IRETETH), 41222 Larissa, Greece

## Abstract

Recurrent urinary tract infections (UTI) have been considered potential triggers of primary biliary cirrhosis (PBC), an autoimmune cholestatic liver disease characterised by progressive destruction of intrahepatic bile ducts. Additional support for the link made between PBC and UTI was based on early observations of recurrent episodes of bacteriuria in female patients with PBC. A series of large epidemiological studies demonstrated a strong correlation between recurrent UTI and PBC, initiating a series of studies investigating the role of *Escherichia coli* (*E. coli*, the most prevalent organism isolated in women with UTI) as a trigger of PBC. Immunological evidence of B- and T-cell cross-reactive responses implicating PBC-specific autoantigens and *E. coli* mimics have been clearly demonstrated, adding support to the notion that *E. coli* is a potential infectious inducer of PBC in susceptible individuals. One of the major limitations in proving the *E. coli*/PBC association was the lack of reliable *E. coli*-infected animal models of PBC. This review provides an overview of the evidence linking this infectious agent with PBC and discusses the pros and cons of a recently developed *E. coli*-infected animal model of PBC.

## 1. Introduction

Primary biliary cirrhosis (PBC) is an autoimmune cholestatic liver disease characterized by high-titre antimitochondrial antibodies (AMA), as well as disease-specific antinuclear antibodies (ANA) [1, 2]. The presence of AMA is considered pathognomonic for PBC, as serum AMA positivity predicts disease development in asymptomatic individuals [3]. The natural course of PBC is generally slow, although the disease course is unpredictable. Over the years, the progression of the disease leads to the inflammatory destruction of small intrahepatic bile ducts, which progresses to fibrosis, cirrhosis, and eventual liver failure [1].

As the disease overwhelmingly affects middle-aged females frequently complaining from recurrent urinary tract infections (UTI),* Escherichia coli* (*E. coli*) has been postulated as a potential trigger for the development of the disease [4, 5]. Epidemiological, immunological, and microbiological data have provided strong evidence in support of the pathogenic link between this bacterium and the disease [6–18]. These data are comprehensively discussed elsewhere and will not be mentioned in the present report.

AMA are directed against components of the 2-oxoacid dehydrogenase complexes, which mainly recognise the E2 subunit of the pyruvate dehydrogenase complex (PDC) in 90% of cases [2, 19]. In 20–70% of cases, the E2 subunits of branched-chain 2-oxoacid dehydrogenase complex (BCOADC) and 2-oxoglutarate dehydrogenase complex (OGDC) are also targeted, while the E1*α* and E1*β* subunits of PDC have been identified as subdominant autoantigenic targets [2, 19]. Anti-PDC-E2 antibodies cross-reactively recognize PDC-E3 binding protein (PDC-E3BP), formerly known as PDC-X [2, 19].

**Figure 1:**
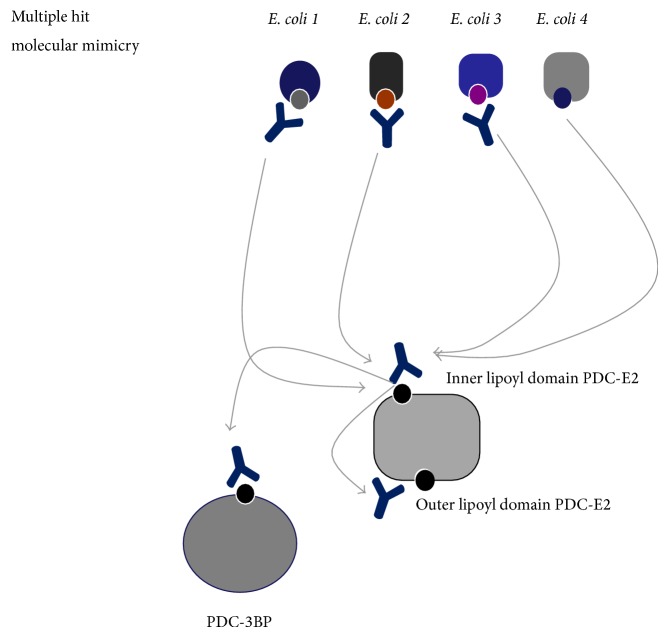
A microbial/self-multiple hit mechanism of molecular mimicry including several primary biliary cirrhosis- (PBC-) specific autoepitopes and their* E. coli* mimics (numbered 1–4 corresponding to those with reactivity depicted in Table 2) is likely involved in the induction of antimitochondrial antibody (AMA) responses in PBC. We propose that a multiple hit mechanism of intra- and intermolecular mimicry is operated at the B-cell level. This mechanism involves several mimics from various* E. coli* proteins which share a high degree of homology with the major mitochondrial autoepitope located at the inner lipoyl domain of the pyruvate dehydrogenase complex E2 subunit (PDC-E2). Urinary tract infections initiate an immune response against the* E. coli* mimics which in turn cross-react with the human mitochondrial autoantigens (arrows). Autoantibody responses against the human ILD PDC-E2 autoepitope initiate cross-reactive response to the mimicking sequences of the outer lipoyl domain of PDC-E2 and its mimic on the E3 binding protein (E3BP) of PDC (arrows). This multiple hit intra- (between the inner and the outer lipoyl domain of the same protein) and inter- (between different self-proteins and microbial proteins) mechanism of molecular mimicry may explain several specificities of the multiantigen specificities seen in PBC, as well as in other autoimmune diseases.

The exact mechanisms that lead to the loss of immunological tolerance to mitochondrial autoantigens (such as PDC-E2) are unclear [20–22]. Nevertheless, specific infectious agents including* E. coli* (the most frequent pathogen for recurrent urinary tract infection in women), as well as* Novosphingobium aromaticivorans* [23] and* Lactobacillus delbrueckii*, have been considered the most significant infectious triggers, but these have been studied more extensively. The mechanism of molecular mimicry and cross-reactivity involving* E. coli* and human PDC-E2 epitopes (or other mitochondrial antigens) has been considered the most likely trigger of the initiation of* E. coli*-associated antimitochondrial immune responses (Figure 1) [24, 25]. In fact, strong evidence regarding CD4 T-cell cross-recognition of* E. coli* and human mitochondrial autoantigens has been obtained over the years, further supporting the concept of molecular mimicry as the driving force of the immunological breakdown characteristic of PBC. An overview of the evidence provided thus far on immunological studies investigating the role of molecular mimicry is given below.

As for other diseases, external support of the pathogenic association between* E. coli* and PBC could stem from studies on animal models of PBC based on* E. coli*-infected mice (Table 1). These long awaited animal models of PBC have now been developed, and the present review discusses the major features of these mice and their relevance to the human disease [26].

## 2. *E. coli*, Molecular Mimicry, and PBC

Anti-PDC-E2 antibody positive PBC cases recognise* E. coli* PDC-E2, but this reactivity is 100-fold lower compared to that against mammalian PDC-E2 [27]. This is also the case for cross-recognition of OGDC-E2, another OADC-E2 subunit [28]. At the B-cell epitope level, antibodies against PDC-E2_212–226_, which is the core antibody autoepitopic region of human PDC-E2, do not appear to cross-react with the corresponding PDC-E2 sequences of* E. coli*. Our group has proposed that the lack of humoral cross-reactivity may be due to differences between human and* E. coli* PDC-E2 on the structural level, which make antigenic cross-recognition impossible. Our hypothesis was a valid one, as epitope prediction analysis has shown that the extent of 3D mimicry between* E. coli* and human PDC-E2 is not sufficient enough to initiate cross-reactive immune responses [29]. Nevertheless, the core epitopic region of the B-cell PDC-E2 epitope overlaps with the immunodominant CD4 and CD8 T-cell epitope, and HLA class II restricted motifs shared by the human and* E. coli* PDC-E2 do exist [25]. Experimental findings support the presence of cross-reactive CD4 and CD8 T-cell responses between human and* E. coli* PDC-E2 [10, 11, 25, 30]. Shimoda and colleagues have developed CD4 T-cell lines with specificity for the disease-specific human PDC-E2 autoepitope (GDLLAEIETDKATI) and its* E. coli* homologue PDC-E2 (EQSLITVEGDKASM). That group also demonstrated that these two cross-react at the CD4 T-cell level. Shimoda and colleagues have delineated the fine specificity of these T-cell lines and demonstrated that the ExDK motif shared by human and* E. coli* PDC-E2 is of paramount importance for epitope recognition [10, 11]. These reports also demonstrated that T-cell lines specific to the human PDC-E2 autoepitope developed from PBC patients peripheral blood mononuclear cells, or liver infiltrating cells, can proliferate in the presence of an* E. coli* OGDC-E2 peptide containing the ExDK motif [10, 11]. Amongst 16 T-cell clones specific for* E. coli* OGDC-E2 peptide, 13 could respond to human OADC-E2 autoepitopes from PDC-E2, OGDC-E2, and BCOADC-E2 [12].

Several investigators have pointed out that motifs shared by* E. coli* and human OADC proteins are to be expected in view of the highly conserved nature of OADC (Table 2). However, previous studies have also shown that* E. coli* sequences not related to PDC-E2, BCOADC-E2, or OGDC-E2 are highly homologous to PDC-E2_212–226_ and some are also cross-reactive targets of antibodies specifically present in patients with PBC, particularly those with recurrent episodes of UTI [10, 15]. Among the six* E. coli* mimics, only 2 originating from the ATP-dependant helicase hrpA, and the periplasmic binding protein cross-recognised, and their respective peptides were able to absorb out reactivity to human PDC-E2_212–226_ [15]. As four other mimicking sequences were totally unreactive, we speculated that reactivity to ATP-dependant helicase hrpA is not epiphenomenal and has potential significance for the pathogenesis of PBC [15, 22, 29].

Two independent studies in PBC cohorts from Spain and the UK have demonstrated a disease-specific presence of antibodies against a short 18-meric sequence from the ATP-dependant Clp protease (ClpP_177–194_). Antibody reactivity against this peptide was found in one-third of the cases with PBC but in less than 4% of the controls tested [8, 16]. This finding was of potential interest for several reasons. First, a three-dimensional model of the* E. coli* ClpP clearly demonstrates that this region of ClpP_177–194_ is exposed on the surface of the molecule and as such could be an easy target for antibody binding, further explaining its high affinity for the respective antibodies [29]. Peculiarly enough, ClpP is in a complex with the regulatory ATP-binding subunit X of the* E. coli* Clp complex ClpX, and a peptide of ClpX shares a striking homology with the dominant human PDC-E2_212–226_ epitope [15]. However, antibody testing of the ClpX mimic did not reveal any evidence of significant humoral response in patients with PBC [15]. Thus, despite being a mimic,* E. coli* ClpX is not an antibody target. This is in contrast to ClpP that is not homologous but is targeted by antibodies. We provided an explanation for this paradoxical finding and suggested that recognition of ClpP/ClpX by PBC-specific antibodies leads to internalization of the ClpP and subsequent B-cell presentation of the Clp X peptide to CD4 helper T-cells [15, 29]. To this end, we have provided preliminary data demonstrating the presence of strong CD4 T-cell responses to* E. coli* ClpX. ClpP was not a T-cell target [29]. The biological significance of these findings requires external validation and further investigation as the ClpP epitope is highly conserved amongst bacteria and the respective ClpP homologue from other bacteria is also a target of cross-reactive responses in PBC women with recurrent UTI [16].

According to these scenarios, recurrent UTI leads to an initiation of anti-*E. coli* and cross-reactive PBC-specific AMA responses and subsequently to liver disease. As PBC is also characterized by disease-specific ANA, evidence must be sought in search for molecular mimics between* E. coli* proteins and human nuclear autoantigens of PBC-specific ANA [14]. It is of interest that the great majority of women with recurrent UTI but no liver disease, who are AMA positive, also react with sp100. None of the AMA negative women in this group showed anti-sp100 antibody reactivity [14]. These data show that antibody reactivity to sp100 correlates with AMA positivity and a history of recurrent UTI. The fact that women without PBC but with a history of recurrent UTI also have anti-sp100 but not anti-gp210 antibodies led us to speculate that* E. coli* is linked to PBC and that this infectious agent is most likely a trigger of AMA and sp100-specific ANA (rather than gp210) production. This scenario would fit with the finding that gp210 and sp100 autoantibodies rarely coexist in the same patient, which suggests that different triggers may account for their initiation during the development of the disease.

## 3. *E. coli*-Based Animal Models of PBC

There is no doubt that any attempt to consider* E. coli* as a pathogen of PBC has to find experimental support on the basis of a reliable animal model of the disease. This model would have to be induced after exposure of the animals to uropathogenic strains of* E. coli*. As for any other experimental model of a given disease, the animal model would have to be characterized by high reproducibility in terms of disease frequency and prolonged disease maintenance [26]. The immunological and histopathological features of the affected animals would have to show high resemblance with that of the human disease (Table 1). The animal would have to be presented with AMA targeting epitopic regions corresponding to those of the human mitochondrial autoantigens, as well as PBC-specific ANA against nuclear body and nuclear envelope antigens that is similar to those noted in a significant proportion (25–50%) of PBC women. In addition, AMA and ANA seen in sera from affected animals would need to show fine specificity comparable to that noted in women with PBC. Ideally, the fine specificity of cellular immune responses against mitochondrial autoantigens noted in mice would match those noted in humans. In terms of clinical relevance, nonspecific symptoms like those of pruritus and fatigue observed in a considerable proportion of women with PBC could also be seen in experimentally induced PBC. PBC is characterized by the cocurrent presence of other autoimmune diseases (mainly sicca symptomatology and autoimmune thyroiditis); thus, an ideal experimental model would be that showing extrahepatic features similar to those of women affected with PBC.

Practically speaking, all animal models of diseases including those developed for autoimmune diseases such as PBC lack some of the key features of the disease under investigation. Nevertheless, work performed over the years in the field of experimental PBC has led to the development of animal models closely resembling the human condition, including most if not all of the typical features of the disease (reviewed in [26]).

In 2008, Palermo published in the form of an abstract a monography reporting the development of an* E. coli*-based experimental model of PBC [9]. According to the author, the animals used were seven-week-old female C57Bl/6 mice receiving transurethral inoculations with uropathogenic* E. coli*. Four months after the original inoculation, the mice developed histological and immunological features of PBC. The histological features included intrahepatic bile-duct destruction, granuloma formation, and lymphocyte infiltration [9]. AMA were induced at early time points following inoculation. This model, which resembles early PBC, has never been published in a form of a full-length paper, and a detailed analysis of the features of the affected animals has not been performed.

A recent study from the Gershwin's group has described a novel model of* E. coli* induced PBC [31]. NOD.B6 Idd10/Idd18 mice were infected with* E. coli* [31]. These mice were followed up for several weeks following infection and histological as well as immunological features were assessed in great detail.

## 4. AMA Responses in* E. coli*-Infected PBC Animals

In the study by Gershwin's group, the animals developed AMA reaching their peak four weeks after the infection, and subsequently their concentration diminished to levels comparable to those noted in mice infected with* Novosphingobium aromaticivorans* [31]. While AMA reactivity in the* E. coli*-infected mice is stronger than that noted in* N. aromaticivorans*, this reactivity is weaker than that seen in serum samples from women with PBC or in other animal models of PBC. These include the dominant negative transforming growth factor- (dnTGF-) *β*RII mice and xenobiotic 2-octynoic acid bovine serum albumin (BSA) conjugate-immunized mice [13, 32].

Antibody measurements showed reactivity against PDC-E2 and OGDC-E2 but not against BCOADC-E2 [31]. The* E. coli*-infected mice developed histological features such as portal inflammation and granuloma formation long after the induction of AMA responses. Thus, while AMA reached their peak at 4 weeks after infection, histological features presented at 26 weeks [31]. This finding is of interest, as several investigators believe that AMA directly or indirectly participate in the destruction of small bile ducts. These findings do not support the notion believed by many investigators that AMA (and in general autoantibodies) are secondary phenomena following the destruction of biliary epithelial destruction and the release of cryptic mitochondrial autoantigens. PBC-specific ANA were not tested in these mice.

## 5. Histological Features of the* E. coli*-Based PBC Animal Model

Histological assessment of the mice showed portal inflammation, granuloma formation, and biliary cell damage in the livers of both* N. aromaticivorans* and* E. coli*-infected mice. Immunohistochemical staining for CK19 showed biliary epithelial cells among lymphoid aggregates and a diverse degree of biliary cell damage [31]. The heterogeneous form of cell destruction involved biliary epithelial cells with moderate to severe inflammatory cell infiltration and cell destruction in some portal tracts, while neighboring tracts were almost undamaged showing only a mild lymphoid aggregation [31].

The results of this study show that* E. coli* infection can inflict PBC-mimicking biliary epithelial cell damage in the biliary disease-prone NOD.B6-Idd10/Idd18 mice.

This model may shed light on the understanding of the long-presumed association of recurrent UTI with the development of primary biliary cirrhosis.

## Figures and Tables

**Table 1:** Immunological and histological features of patients with primary biliary cirrhosis (PBC) and PBC-resembling experimental *E.  coli*-infected NOD.B6 Idd10/Idd18 mice.

	PBC	*E. coli*-infected NOD.B6 Idd10/Idd18 mice
Immunological features		
**AMA**	Yes	Yes
Anti-PDC-E2	Yes	Yes
Anti-OGDC-E2	Yes	Yes
Anti-BCOADC-E2	Yes	No
**ANA**	Yes	Not tested
Histology		
Portal infiltration	Yes	Yes
Granuloma formation	Yes	Yes
Bile-duct destruction	Yes	Yes

PDC: pyruvate dehydrogenase complex; OGDC: 2-oxoglutarate dehydrogenase complex; BCOADC: branched-chain 2-oxoacid dehydrogenase complex.

**Table 2:** Amino acid similarities between *E.  coli* and self-proteins in patients with primary biliary cirrhosis (PBC).  There are three major targets of cross-reactive autoantibodies directed against an epitope located at the inner lipoyl domain (ILD) of human pyruvate dehydrogenase E2 complex (PDC-E2), a cross-reactive one at the outer lipoyl domain (OLD), and one mimic on the PDC E3-binding protein (E3BP). Amongst six PDC-E2 mimics originated from various *E.  coli* proteins, four are targets of cross-reactive responses while three are unreactive, including the *E.  coli* PDC-E2 mimic which is a weak target. Amino acids appear in standard single letter code. Sequence alignment has been performed using the BLAST2p protein-protein comparison programmes. + indicates conserved or semiconserved substitutions.

															Protein	Identity	Similarity	Reactivity
G	Q	*A *	M	*V *	**D**	**L**	**L**	**A**	**E**	Y	**E**	K	V	G	*E. coli* nitrate reductase 2	6	53% (8/15)	No
**K**	*A *	**S**	**E**	**G**	*E *	**L**	**L**	**A**	*Q *	*V *	**E**	*P *	*E *	D	*E. coli* ATP-dependent Clp-X	8	93% (14/15)	No
**K**	*V *	*A *	A	E	*Q *	S	**L**	*I *	T	*V *	**E**	G	**D**	**K**	*E. coli * PDC-E2	5	73% (11/15)	Weak

**K**	**L**	**S**	**E**	**G**	**D**	**L**	**L**	**A**	**E**	**I**	**E**	**T**	**D**	**K**	**Human PDC-E2 ILD**			

L	*M *	*T *	*D *	**G**	I	**L**	**L**	**A**	**E**	**I**	*Q *	Q	**D**	*R *	*E. coli* ATP-dependent helicase	7	80% (12/15)	Yes
G	Y	*A *	*Q *	*S *	G	**L**	**L**	**A**	**E**	**I**	T	*P *	**D**	**K**	*E. coli* periplasmic maltose-binding	7	73% (11/15)	Yes
D	*A *	*A *	V	E	**D**	**L**	**L**	**A**	**E**	*V *	S	Q	P	**K**	*E. coli* fatty acid oxidation-*α*	6	60% (9/15)	Yes
L	*A *	*T *	L	**D**	**D**	**L**	**L**	**A**	**E**	**I**	G	L	G	*N *	*E. coli* (P) ppGpp synthetase II	6	60% (9/15)	Yes

A	*V *	**S**	A	**G**	**D**	*A *	**L**	C	**E**	**I**	**E**	**T**	**D**	**K**	**Human PDC-3BP**	**10**	80% (12/15)	Yes (strong)

## Abbreviations

AMA: Antimitochondrial antibody
ANA: Antinuclear antibody
BEC: Biliary epithelial cell
*E. coli*: * Escherichia coli*
OADC: Oxoacid dehydrogenase complex
OGDC: 2-Oxoglutarate dehydrogenase complex
PBC: Primary biliary cirrhosis
PDC: Pyruvate dehydrogenase complex
UTI: Urinary tract infection.
